# The Applications and Future Prospects of Organ Medicine

**DOI:** 10.1002/hcs2.70090

**Published:** 2026-08-03

**Authors:** Jiayi Zhang, Youlu Liu, Yuexin Li, Rican Hu, Runbing Mo, Yuyi Zhang, Guangliang Huang, Xiaoshun He

**Affiliations:** ^1^ Organ Transplant Center, The First Affiliated Hospital Sun Yat‐sen University Guangzhou China; ^2^ Guangdong Provincial Key Laboratory of Organ Medicine Guangzhou China; ^3^ Guangdong Provincial International Cooperation Base of Science and Technology (Organ Transplantation) Guangzhou China; ^4^ Department of Medical Ultrasonics, Institute of Diagnostic and Interventional Ultrasound, The First Affiliated Hospital Sun Yat‐sen University Guangzhou China; ^5^ Zhongshan School of Medicine Sun Yat‐sen University Guangzhou China; ^6^ Department of Medical Ultrasonics Yuxi Zhongshan Hospital Yuxi China

**Abbreviations**
IOP: isolated organ perfusion
IOPT: isolated organ perfusion treatment
MP: machine perfusion
NMP: normothermic machine perfusion

## 1 Introduction

Medical research spans multiple levels—from population and individual studies to cellular and molecular investigations—encompassing disciplines such as epidemiology, clinical medicine, cell biology, and molecular medicine. However, the organ level remains underexplored due to the historical lack of technology capable of maintaining isolated organs under physiological conditions ex situ [1]. This gap has hindered a direct and precise understanding of organ function and disease mechanisms, often leading to systemic therapies with unavoidable side effects.

Throughout scientific history, transformative devices have catalyzed paradigm shifts. The steam engine and telescope, for example, revolutionized industry and astronomy, respectively. Similarly, ex situ machine perfusion (MP), initially developed for organ transplantation, now offers a novel platform for advancing medical science. Normothermic machine perfusion (NMP) in particular simulates in situ circulation, maintaining organ viability and functionality outside the body. This enables controlled investigation of organ physiology, disease pathogenesis, and the development of targeted therapies.

**Figure 1:**
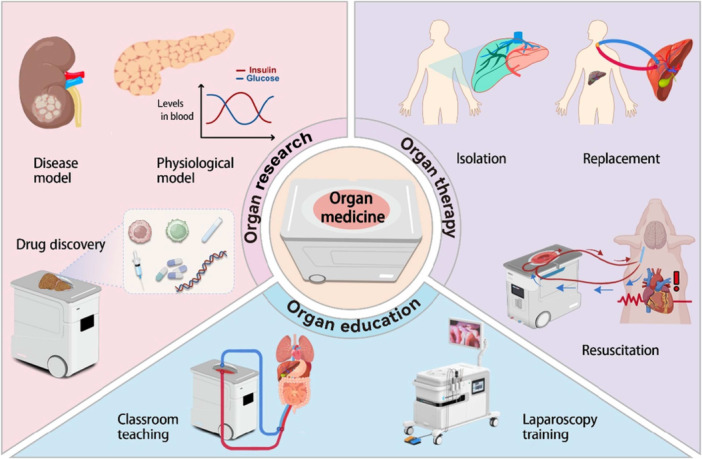
Summary of organ medicine. The concept of organ medicine has demonstrated its importance across three dimensions: organ therapy, organ research, and organ education. These approaches span multiple organs, including the liver, brain, heart, kidney, and lung. Organ therapy involves diverse approaches for the repair and regeneration of a single organ. It also involves the replacement and isolation of a target organ within the systemic circulation. Organ research is used to reveal the physiological functions of organs, providing a profound insight into the disease process at the organ level. Disease organ models, arising from this research, serve as promising models for new drug discovery and testing. In the field of organ education, perfused organs can be used in classroom teaching and laparoscopy training.

Dr. Xiaoshun He's group has recently proposed a new concept of Organ Medicine (Figure 1), namely, investigation and treatment of diseases at an organ level with the help of isolated or ex situ machine perfusion technology [2]. Herein, we write this editorial with the aim of engaging the wider scientific community in this cutting‐edge area. We will explore the potential use of this concept in both biology and medicine. We will discuss the current status and future directions of Organ Medicine in the aspects of organ research, organ therapy, as well as organ education.

## 2 Organ Research

Ex situ MP serves not only as a tool for organ repair but also as a versatile research platform. It allows detailed study of organ physiology and pathophysiology, inter‐organ interactions, and provides a stable model for drug discovery and testing.

### 2.1 Organ Physiological Function

The use of isolated organ perfusion (IOP) to study physiological function dates back over a century. Using animal organs, IOP enabled discoveries in inflammatory responses and metabolic regulation [3, 4]. It also facilitated the integration of advanced imaging techniques [5, 6], such as multiphoton microscopy and hyperpolarized metabolic imaging, offering dynamic and metabolic insights into organ function.

However, IOP has limitations, including non‐physiological perfusion conditions and the use of acellular perfusates. In contrast, NMP provides a more accurate simulation of the in situ environment by maintaining normothermia, physiological pressures, oxygenation via integrated oxygenators, and using blood‐based perfusates (Table 1). Real‐time monitoring and software‐controlled algorithms further enhance physiological relevance.

**Table 1:** Differences between IOP and NMP.

Parameter	IOP	NMP
Perfusion pressure	Pressure uncontrolled with only flow controlled (mostly low flow with low pressure)	Physiological perfusion pressure controlled
Perfusion method	Pulsatile or constant perfusion	Pulsatile perfusion
Temperature	Hypothermic	Normothermic
Oxygenation	Gassed or oxygenated perfusate (gas enrichment into perfusate reservoir before circulation) using glassware with bubble oxygenator or rotating disc oxygenator	Oxygenation systems using real‐time extracorporeal membrane oxygenation
Perfusate composition	Cell‐free isotonic, isotonic solutions	Blood‐based solutions
Monitoring system	No monitoring system	Real‐time blood‐gas monitoring

Abbreviations: IOP, isolated organ perfusion; NMP, normothermic machine perfusion.

While single‐organ perfusion is informative, it cannot fully replicate the complexity of multi‐organ interactions in situ. Multi‐organ perfusion models, such as combined liver‐kidney circuits, have demonstrated synergistic benefits including enhanced adenosine triphosphate production, reduced oxidative stress, and moderated inflammatory responses. More comprehensive models, such as the abdominal multiple organ block perfusion system, offer unprecedented platforms for studying inter‐organ communication, though practical constraints often limit simultaneous perfusion to one or two organs [7].

### 2.2 Disease Organ Model

Current disease models include cell cultures, animal models, and organoids. While valuable, each has limitations: cell models lack tissue‐level complexity; animal models may not fully replicate human physiology; and organoids often resemble fetal rather than adult tissues and lack functional vasculature and immune components.

Disease organ models, enabled by NMP, overcome many of these shortcomings. Organs discarded from transplantation or resected during surgery can be perfused ex situ, preserving their native cellular diversity, vascular architecture, and immune microenvironment. Such models have been successfully established for infectious diseases [8] (e.g., SARS‐CoV‐2 in lung models) and cancers [9] (e.g., liver tumors), providing a physiologically relevant platform for pathogenesis studies and therapeutic testing.

Multi‐organ disease models further allow investigation of systemic conditions. For example, liver‐spleen perfusion models have been used to study *Klebsiella pneumoniae* infection, revealing previously unrecognized intracellular behavior of the pathogen [10]. These advances underscore the potential of ex situ organ models in bridging the gap between conventional models and clinical translation.

### 2.3 Drug Discovery and Testing

Traditional drug discovery relies heavily on cell lines and animal models, but high failure rates in late‐stage clinical trials often reflect poor translation from these systems to humans [11]. Organoids have improved relevance through their human origin and three‐dimensional structure, yet challenges remain in reproducibility, scalability, and physiological maturity.

Disease organ models offer a complementary approach. While not suited for high‐throughput screening, they provide a unique platform for evaluating drug absorption, distribution, metabolism, excretion, and efficacy in a near‐native human organ environment. Integrated workflows combining organoids, animal models, and ex situ human organ perfusion may accelerate drug development and reduce attrition in clinical phases [8].

### 2.4 Future Directions

Future efforts should focus on expanding the repertoire of disease organ models beyond the liver and lungs, optimizing protocols for using surgical specimens, and establishing standardized evaluation criteria for drug testing. Reliable, scalable organ models will enhance our understanding of disease mechanisms and improve the predictive accuracy of therapeutic interventions.

## 3 Organ Therapy

### 3.1 Isolated Organ Perfusion Treatment (IOPT)

Systemic drug administration often leads to off‐target effects and organ toxicity. IOPT allows targeted delivery of therapeutics to a specific organ while isolating it from systemic circulation. This approach minimizes side effects, avoids hepatic metabolism, and enables precise control of the local microenvironment.

Clinically, IOPT has been applied in isolated limb infusion for melanoma [12], isolated hepatic perfusion for liver metastases, and percutaneous hepatic perfusion [13, 14]. Recent innovations include intraoperative renal perfusion during aortic aneurysm repair [15], limb perfusion during oncological resection, and targeted chemotherapy delivery to hepatic tumors using in situ MP [13, 16]. These cases illustrate the potential of IOPT to integrate with surgical procedures for organ‐specific treatment.

Future development should establish standardized perfusion protocols for different organs, optimize drug dosing and delivery, and extend the approach to gene and cell‐based therapies. Collaboration across disciplines will be essential to advance IOPT as a modality in precision medicine.

### 3.2 Extracorporeal Organ Support

Extracorporeal organ support aims to temporarily replace or assist the function of a failing organ. Extracorporeal liver perfusion, for example, has been used to bridge patients with acute liver failure to transplantation [17]. Advances in xenogeneic organ sourcing and MP technology may expand the availability and efficacy of such support systems [18].

Cross‐circulation—connecting a patient's organ to a healthy host or a genetically engineered animal—offers another innovative platform for organ repair and recovery. Studies have demonstrated the feasibility of using porcine cross‐circulation to restore injured human lungs, highlighting a transient and immunologically manageable exposure to xenoantigens [19, 20].

### 3.3 Extracorporeal Liver Support for Brain Resuscitation

Global cerebral ischemia, as occurs in cardiac arrest, lacks effective treatments [21, 22]. MP technology has shown promise in restoring cellular and metabolic functions in the brain after circulatory death. Studies using the BrainEx system demonstrated revival of microcirculation and neural activity in porcine brains post‐mortem [21]. Combined brain‐liver perfusion further extended functional recovery and even restored signs of consciousness, underscoring the importance of inter‐organ support in resuscitation.

These findings suggest potential applications in emergency medicine for stroke and cardiac arrest and may eventually challenge current definitions of death and organ procurement timing.

### 3.4 Future Directions

Ex situ MP‐based therapies, including IOPT, extracorporeal support, and resuscitation platforms, hold transformative potential. Future work should aim to adapt these techniques to other organ systems, refine physiological monitoring, and navigate associated ethical and logistical challenges.

## 4 Organ Medical Education

### 4.1 Basic Medical Education

Traditional anatomy education relies on textbooks, atlases, videos, and cadaveric dissection, each with limitations in interactivity, accessibility, and realism. Perfused organs offer a dynamic alternative, preserving natural color, texture, and physiological responsiveness. They provide tactile and visual feedback critical for surgical training, bridging gaps left by virtual simulators and static specimens.

Portable perfusion systems allow organs to be used in diverse educational settings, supporting system‐based curricula and offering real‐time physiological demonstrations. Such models are particularly valuable in laparoscopic and endoscopic training, where tissue handling, bleeding simulation, and emergency management can be realistically replicated.

### 4.2 Laparoscopy and Endoscopy Training

Devices such as the Life‐T100 Pro combine NMP with multi‐organ blocks to create high‐fidelity surgical simulators. These platforms support procedures like cholecystectomy and hepatectomy while maintaining organ viability and permitting real‐time monitoring. Evaluation frameworks incorporating organ vitality metrics and procedural realism, assessed by expert surgeons, can standardize training outcomes and ensure clinical relevance [23].

### 4.3 Future Integration

The integration of artificial intelligence, computer vision, and advanced imaging into organ‐based simulators will further enhance training efficiency and objectivity. A comprehensive ecosystem combining realistic simulation, automated skill assessment, and personalized feedback promises to elevate surgical education and improve patient safety.

## 5 Summary

Organ Medicine represents a paradigm shift beyond transplantology, integrating ex situ perfusion technology into research, therapy, and education. By enabling organ‐level investigation, targeted treatment, and immersive training, this field holds promise to deepen our understanding of human physiology and disease, accelerate therapeutic innovation, and redefine medical practice. Its interdisciplinary nature will further foster convergence with bioengineering, biology, and related sciences, ultimately contributing to improved health outcomes and quality of life.

## Author Contributions

**Jiayi Zhang:** supervision, writing – review and editing, methodology, validation, visualization, formal analysis. **Youlu Liu:** writing – review and editing, visualization, formal analysis, project administration. **Yuexin Li:** software, formal analysis, project administration. **Rican Hu:** formal analysis, writing – original draft. **Runbing Mo:** writing – original draft, software. **Yuyi Zhang:** writing – original draft, software. **Guangliang Huang:** writing – review and editing, supervision. **Xiaoshun He:** funding acquisition, investigation, supervision, resources, data curation, writing – review and editing.

## Ethics Statement

The authors have nothing to report.

## Consent

The authors have nothing to report.

## Conflicts of Interest

The authors declare no conflicts of interest.

## Data Availability

The data that support this article are available from the corresponding author upon reasonable request.
